# Association Between High Sensitivity Cardiac Troponin and All-Cause and Cardiovascular Mortality in Adults at Risk of Non-Alcoholic Fatty Liver Disease: A Cohort Study

**DOI:** 10.5334/gh.1427

**Published:** 2025-04-29

**Authors:** Enfa Zhao, Hang Xie, Yuan Gao, Xiaolin Wen, Bingtian Dong, Chaoxue Zhang

**Affiliations:** 1 Department of Ultrasound, The First Affiliated Hospital of Anhui Medical University, Hefei 230022, Anhui Province, China; 2 Department of Cardiovascular Medicine, The First Affiliated Hospital of Xi’an Jiaotong University, Xi’an 710061, Shaanxi, China

**Keywords:** hs-cTn, myocardial injury, NAFLD, mortality, NHANES

## Abstract

**Objective:**

Cardiovascular disease (CVD) is the leading cause of death among patients with non-alcoholic fatty liver disease (NAFLD). This study investigates the association between high-sensitivity cardiac troponin (hs-cTn) levels and mortality in adults at risk of NAFLD in a representative U.S. population sample.

**Methods:**

Among participants aged 18 years and older in the 1999–2004 National Health and Nutrition Examination Survey, we measured high-sensitivity troponin T using a single assay (Roche) and high-sensitivity troponin I using three assays (Abbott, Siemens, and Ortho). Myocardial injury was identified by elevated levels of hs-cTn. Mortality outcomes were determined through linkage with the National Death Index database, with follow-up until December 31, 2019. A multivariable Cox proportional hazards model was used to evaluate the associations between myocardial injury and mortality in the NAFLD population. Sensitivity analyses were conducted to assess the robustness of the main findings.

**Results:**

A total of 2581 at risk of NAFLD were included in this observational study, with myocardial injury identified in 7.01%. Over a median follow-up of 16.7 years, 937 all-cause deaths occurred, including 319 cardiovascular disease-related deaths. NAFLD individuals with myocardial injury had worse survival rates at 5, 10, and 15 years compared to those without myocardial injury. After adjusting for baseline characteristics, myocardial injury was associated with an increased risk of all-cause mortality (adjusted Hazard Ratio [aHR] 1.785, 95% CI 1.494–2.134, *P* < 0.001) and cardiovascular mortality (aHR 2.155, 95% CI 1.606–2.893, *P* < 0.001).

**Conclusion:**

This large, nationally representative study demonstrates that myocardial injury, defined by elevated hs-cTn levels, is independently associated with increased all-cause and cardiovascular mortality risks in the adult population at risk of NAFLD in the United States. This association persisted after adjusting for various factors and in patients without pre-existing cardiovascular disease. The Siemens hs-cTn I assay demonstrated the strongest association with all-cause mortality. These findings highlight the potential of hs-cTn as a valuable prognostic marker in NAFLD patients, even in those without clinically apparent cardiovascular disease. Routine hs-cTn assessment may aid in risk stratification and guide targeted interventions to reduce mortality risk in this population.

## 1. Introduction

Non-alcoholic fatty liver disease (NAFLD) is a condition characterized by the accumulation of fat in the liver in individuals who consume little to no alcohol. It has become a major public health issue worldwide, with an estimated global prevalence of around 25% (1,2). NAFLD is closely linked to obesity, type 2 diabetes, and metabolic syndrome and is considered the hepatic manifestation of this condition (3). While liver-related complications are a major concern in NAFLD, cardiovascular disease (CVD) remains the leading cause of mortality among these patients (4). This highlights the crucial importance of developing effective cardiovascular risk stratification tools for the NAFLD population.

High-sensitivity cardiac troponin (hs-cTn) assays have emerged as powerful biomarkers for detecting subclinical myocardial injury and predicting adverse cardiovascular outcomes (5,6). These assays can detect troponin levels well below the 99th percentile upper reference limit, allowing for the identification of individuals at risk for future cardiovascular events (7). Recent studies have demonstrated the prognostic value of hs-cTn in various populations, highlighting its potential as a flexible biomarker for cardiovascular risk stratification. In the general population, it was found that elevated levels of hs-cTnT and hs-cTnI were independently associated with increased all-cause and cardiovascular mortality, with hazard ratios (HR) ranging from 1.10 to 1.31 per standard deviation increase in log-transformed hs-cTn levels (8). Similarly, in patients with established cardiovascular disease, all four hs-cTn assays (Roche hs-cTnT and three hs-cTnI assays) showed significant associations with both all-cause and cardiovascular mortality, with HRs ranging from 1.31 to 2.16 per standard deviation increase (9). Furthermore, studies in specific patient populations, such as those with chronic kidney disease or diabetes, have also demonstrated significant associations between hs-cTn levels and adverse cardiovascular outcomes (10,11). These findings collectively highlight the strong prognostic capabilities of hs-cTn across various clinical settings and patient populations.

While liver-related complications are a major concern in NAFLD, CVD remains the leading cause of mortality among these patients. Recent meta-analyses have demonstrated that NAFLD is associated with a 45% higher risk of fatal or non-fatal CVD events (HR 1.45, 95% CI 1.31–1.61), with this risk markedly increasing with disease severity, particularly in advanced fibrosis (HR 2.50, 95% CI 1.68–3.72) (12). Given this substantial cardiovascular burden in NAFLD patients, early identification of individuals at highest risk for adverse cardiovascular outcomes is crucial for targeted preventive interventions. This study aims to address this knowledge gap by examining the association between elevated hs-cTn levels, indicative of myocardial injury, and both all-cause and cardiovascular mortality in a large, nationally representative cohort of U.S. adults at risk of NAFLD. By utilizing data from the National Health and Nutrition Examination Survey (NHANES) and comparing multiple hs-cTn assays, we seek to provide comprehensive insights into the prognostic value of these biomarkers in the NAFLD population.

## 2. Materials and Methods

### 2.1 Study population

This study utilized data from the National Health and Nutrition Examination Survey (NHANES), a comprehensive, cross-sectional population-based study conducted by the National Center for Health Statistics of the Centers for Disease Control and Prevention (CDC) in the United States. NHANES employs a complex, multi-stage probability sampling design to assess the health and nutritional status of non-institutionalized civilian adults and children in the U.S. The survey, conducted continuously in two-year cycles since 1999, comprises interviews, physical examinations, and laboratory tests administered by medical professionals at mobile screening centers. The NHANES protocol was approved by the Institutional Review Board of the National Center for Health Statistics through protocol #98–12, and all participants provided written informed consent. The study complies with the Declaration of Helsinki, and the data are publicly available at https://www.cdc.gov/nchs/nhanes.

From the initial 17,061 subjects aged 18 years and older in the NHANES 1999–2004 dataset, we identified 3,754 potential participants with a United States Fatty Liver Index (USFLI) ≥ 30, indicating the presence of non-alcoholic fatty liver disease (NAFLD). We then excluded participants for the following reasons: pregnancy (n = 930), missing cardiac troponin data (n = 3,595), insufficient data to calculate USFLI (n = 6,683), and lack of follow-up information (n = 6). To focus specifically on NAFLD, we further excluded participants with other potential causes of steatohepatitis, including hepatitis B or C infection (n = 61), use of steatogenic medications (n = 255), and those identified as having excessive alcohol consumption (defined as more than four alcohol units per day for men and more than three alcohol units per day for women) (n = 587). After applying these exclusion criteria, our final analytic sample consisted of 2,851 participants, representing 41,828,861 US adults at risk of NAFLD (Figure S1).

### 2.2 Study sample

High-sensitivity cardiac troponin concentrations were measured in stored serum samples at the University of Maryland School of Medicine between 2018 and 2020. The majority (93%) of these samples had never undergone a prior freeze-thaw cycle. Four different hs-troponin assays were utilized: hs-troponin T was measured using the Roche Cobas e601 with Elecsys reagents, while hs-troponin I was measured using three different assays – Abbott ARCHITECT i2000SR, Siemens Centaur XPT, and Ortho Vitros 3600. To ensure the reliability of the measurements, the inter-assay coefficients of variation were carefully monitored and reported for each assay at various concentration levels. All measurements were performed according to the manufacturers’ instructions and in compliance with standard laboratory procedures to ensure the highest quality and consistency of results.

### 2.3 Diagnosis of NAFLD

The Fatty Liver Index (FLI) is a non-invasive scoring system used to predict the presence of non-alcoholic fatty liver disease (NAFLD) (13). It is calculated using a combination of body mass index (BMI), waist circumference (WC), triglyceride levels (TG), and gamma-glutamyl transferase (GGT): FLI = (e0.953 × Ln (TG) +0.139 × BMI+ 0.7 18 × Ln(GGT)+0.053 × WC-15.745) ÷ (1 +e0.953 × Ln (TG) +0 .139 × BMI + 0.718 × Ln(GGT) + 0.053 × WC-15.745) × 100. TG (mg/dl) and GGT (U/L) were derived from laboratory test information, and BMI (kg/m2) and WC (cm) were obtained from physical examination information. Hepatic steatosis was defined as a fatty liver index (FLI) ≥ 30 (13). NAFLD diagnosis was established when the FLI was 30 or higher, after excluding other potential causes of chronic liver disease and ruling out excessive alcohol consumption. While FLI ≥ 60 is widely used to rule in fatty liver disease, we chose FLI ≥ 30 as our primary threshold to capture a broader spectrum of NAFLD, including early stages, while maintaining adequate statistical power for our analyses (14).

#### Assessment of outcomes

Mortality outcomes were determined through linkage with the National Death Index database, with follow-up through December 31, 2019. The primary outcome was all-cause mortality. CVD mortality, a secondary outcome, was ascertained according to the recorded cause of death using International Classification of Diseases, 10th version (ICD-10) codes. Specifically, CVD mortality was defined as death resulting from heart diseases (ICD-10 codes I00-I09, I11, I13, I20-I51) and cerebrovascular diseases (ICD-10 codes I60-I69). The vital status of participants was ascertained through a probabilistic match between NHANES personal identifiers and death certificate records. This probabilistic matching algorithm has been validated by the National Center for Health Statistics, demonstrating a sensitivity of 98.5% and a specificity of 99.4% in mortality ascertainment (15).

### 2.4 Assessment of covariates

Comprehensive information on demographic, lifestyle, and health-related characteristics was collected through structured questionnaires and physical examinations. Demographic data included age, sex, race/ethnicity (categorized as non-Hispanic White, non-Hispanic Black, Mexican American, other Hispanic, and other Race), education level (less than high school, high school graduate, or above high school), marital status (unmarried or married), and family poverty income ratio. Lifestyle factors encompassed smoking status (never, former, or current smokers), alcohol consumption, and medication use (antidiabetic, antihypertensive, statin, and antiplatelet medications). Trained examiners measured blood pressure, body weight, and height at the mobile examination center. Body mass index (BMI) was calculated as weight in kilograms divided by height in meters squared. Hypertension was defined as mean systolic blood pressure ≥140 mmHg, mean diastolic blood pressure ≥90 mmHg, or use of antihypertensive medication. Diabetes was defined as a previous diagnosis by a healthcare professional, use of antidiabetic medication, or fasting blood glucose ≥126 mg/dL. Fasting venous blood samples were collected in accordance with the quality assurance and quality control protocols established by NHANES. Laboratory measurements included fasting plasma glucose, glycated hemoglobin (HbA1c), alanine aminotransferase (ALT), aspartate aminotransferase (AST), GGT, creatinine, uric acid, blood urea nitrogen, triglycerides, total cholesterol, high-density lipoprotein (HDL) cholesterol, low-density lipoprotein (LDL) cholesterol, and C-reactive protein (CRP). These were assessed using standardized procedures at the Centers for Disease Control and Prevention. Health-related characteristics included a history of malignancy and cardiovascular disease (coronary heart disease, angina, heart attack, stroke, or heart failure). All self-reported information was collected during a computer-assisted personal interview to ensure standardized data collection. Sarcopenia was assessed using dual-energy X-ray absorptiometry (DXA) data from NHANES. DXA scans were conducted on eligible participants aged 8 years and older using a QDR 4500A densitometer (Hologic, Inc., Bedford, MA). Appendicular lean mass (ALM) was calculated by summing the lean tissue mass (excluding bone) of the four limbs. Following the Foundation for the National Institutes of Health (FNIH) criteria, sarcopenia was defined as a binary variable based on the ALM to BMI ratio, with cutoff values of ALM/BMI < 0.789 for males and <0.512 for females (16). The National Center for Health Statistics’ Research Ethics Review Board approved the NHANES protocol, and all participants provided written informed consent, as detailed on the NHANES website (https://www.cdc.gov/nchs/nhanes/irba98.htm).

### 2.5 Diagnosis of Myocardial Injury

Myocardial injury was defined based on sex- and assay-specific thresholds for high-sensitivity cardiac troponin (hs-cTn) levels, in accordance with the Fourth Universal Definition of Myocardial Infarction (UDMI). We used four different hs-cTn assays in this study. The specific thresholds for each assay and the rationale for their selection are detailed in the Statistical analysis section.

Given that participants were at home during data collection, we assumed they did not have acute myocardial ischemia at the time of assessment. This assumption aligns with the chronic subclinical myocardial injury often observed in NAFLD patients. While these thresholds were initially established in general populations, we applied them to our NAFLD cohort as there are currently no NAFLD-specific hs-cTn thresholds available.

### 2.6 Statistical analysis

According to the official NHANES recommendations, survey weights were applied to generate estimates representative of the US adult population. Weights for the three survey cycles from 1999 to 2004 were generated using the recommended NHANES formulas for combining weights across these cycles. Myocardial injury was defined according to the fourth Universal Definition of Myocardial Infarction (UDMI), using sex- and assay-specific 99th percentile upper reference limits for each of the four high-sensitivity cardiac troponin (hs-cTn) assays (17). The specific thresholds were: hs-cTnT >22 ng/L (males) and >14 ng/L (females); hs-cTnI Abbott >35 ng/L (males) and >17 ng/L (females); hs-cTnI Siemens >58 ng/L (males) and >39.6 ng/L (females); and hs-cTnI Ortho >12 ng/L (males) and >9 ng/L (females) (18).

Baseline characteristics were summarized using survey-weighted medians (interquartile ranges) for continuous variables and survey-weighted percentages (95% confidence intervals) for categorical variables. For the Cox proportional hazards models, we tested the proportional hazards assumption using Schoenfeld residuals. The normality of continuous variables was assessed using the Shapiro-Wilk test. For those variables that did not satisfy the assumption of normality (p < 0.05), non-parametric tests, namely the Wilcoxon rank-sum test, were utilized. Differences between groups were assessed using survey-weighted linear regression for continuous variables and survey-weighted Chi-square tests for categorical variables. To address the possibility of collinearity, a collinearity test was conducted when the variance inflation factor exceeded five. Variables with more than 10% missing values were excluded from the model. Kaplan-Meier curves were used to estimate cumulative survival rates at 1, 5, 10, and 15 years for patients with and without myocardial injury, stratified by the specific high-sensitivity cardiac troponin (hs-cTn) assay used. Cox proportional hazards models were employed to estimate hazard ratios (HRs) and 95% confidence intervals (CIs) for the associations between myocardial injury and mortality outcomes. Models were adjusted for a comprehensive set of covariates including age, sex, race/ethnicity, BMI, education level, marital status, history of malignancy, CVD, diabetes mellitus, hypertension, smoking history, statins use, antihypertensive drugs, antidiabetic drugs, antiplatelet drugs, glucose, total cholesterol, diastolic blood pressure, systolic blood pressure, HbA1c (%), ALT; HDL-C; CRP, blood urea nitrogen, and uric acid. We performed several sensitivity analyses to evaluate the robustness of our findings. These analyses included: (1) assessing the associations in patients without a known history of CVD, (2) excluding participants who died within the first two years of follow-up to reduce the likelihood of potential reverse causation, (3) examining the associations for each hs-cTn assay individually (Roche hs-cTnT, Abbott hs-cTnI, Siemens hs-cTnI, and Ortho hs-cTnI), and (4) applying an alternative definition of hepatic steatosis using a FLI cut-off value of 60. These sensitivity analyses were performed to ensure the consistency of our results under different conditions and definitions. All statistical analyses were performed using R software version 4.2.2 (R Foundation for Statistical Computing, Vienna, Austria). We utilized several R packages for specific analyses: ‘survival’ for survival analysis and Cox proportional hazards modeling, ‘rms’ for regression modeling strategies, ‘survey’ for analyzing complex survey designs, and ‘ggplot2’ for creating graphical representations of the data. The ‘EmpowerStats’ software (X&Y Solutions, Inc., Boston, MA) was also used for some statistical analyses. A two-sided P-value <0.05 was considered statistically significant for all analyses.

## 3. Results

### 3.1 Baseline characteristics

A total of 41,828,861 weighted records (2,581 unweighted) representing adults at risk of NAFLD were included in this analysis (Table S1). Among these, 7.01% had myocardial injury and were characterized by older age (71 vs. 47 years, P < 0.001), higher prevalence of cardiovascular disease (37.638% vs. 8.682%, P < 0.0001), and greater comorbidity burden including diabetes mellitus (30.246% vs. 10.940%, P < 0.0001) and hypertension (70.230% vs. 43.543%, P < 0.0001). Detailed baseline characteristics stratified by myocardial injury status are presented in Table S2.

### 3.2 All-cause and cardiovascular mortality

**Figure 1:**
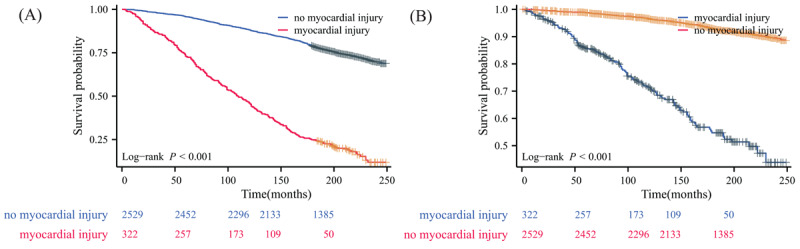
Kaplan–Meier survival curve for patients with non-alcoholic fatty liver disease using any high-sensitivity cardiac troponin assay to define myocardial injury. (A) all-cause mortality; (B) cardiovascular disease mortality.

The median follow-up period for this analysis was 16.6 years (IQR 14.1–18.7). Kaplan-Meier survival curves demonstrated significantly lower survival rates for subjects at risk of NAFLD with myocardial injury compared to those without, for both all-cause and cardiovascular mortality (Log-rank *P* < 0.001 for both). For all-cause mortality (Figure 1A), the cumulative survival rates for patients with myocardial injury were consistently lower at all time points. At one year, the all-cause survival rate was 96.943% for those with myocardial injury versus 99.735% for those without. This difference widened over time, with 5-year survival rates of 74.604% versus 97.212%, 10-year survival rates of 52.734% versus 91.017%, and 15-year survival rates of 32.777% versus 83.871%, respectively (Table 1). The cardiovascular mortality curves (Figure 1B) showed a similar pattern, with a more pronounced difference between the two groups, particularly in the earlier years of follow-up. The Kaplan-Meier curves (Figures S2–S5) display the survival figures stratified by the type of hs-cTn assay used to define myocardial injury.

**Table 1:** Cumulative survival rates for non-alcoholic fatty liver disease patients at 1, 5, 10, and 15 years, categorized by the specific high-sensitivity cardiac troponin test used to identify myocardial injury (unadjusted).


hs-cTn ASSAY/TIME POINT*	WITHOUT MYOCARDIAL INJURY	MYOCARDIAL INJURY

Any hs-cTn assay (weighted)	38897184	2931677

1 year, %	99.735	96.943

5 years, %	97.212	74.604

10 years, %	91.017	52.734

15 years, %	83.871	32.777

hs-cTn T (weighted)	39241902	2586958

1 year, %	99.738	96.535

5 years, %	97.190	71.937

10 years, %	90.942	48.774

15 years, %	83.590	30.238

hs-cTn I Abbott (weighted)	41342518	486343

1 year, %	99.637	91.275

5 years, %	95.869	75.136

10 years, %	88.664	60.257

15 years, %	80.804	36.623

hs-cTn I Siemens (weighted)	41223397	605463

1 year, %	99.604	95.181

5 years, %	95.857	80.008

10 years, %	88.722	61.890

15 years, %	80.922	37.250

hs-cTn I Ortho (weighted)	41342518	486343

1 year, %	99.637	91.275

5 years, %	95.869	75.136

10 years, %	88.664	60.257

15 years, %	80.804	36.623


*All statistical analyses and derived estimates utilize weighted data records

After adjustment for baseline characteristics and comorbidities using Cox regression model, myocardial injury remained significantly associated with increased risk of both all-cause and cardiovascular mortality in NAFLD patients (Table 2). For all-cause mortality, patients with myocardial injury identified by any hs-cTn assay had an adjusted hazard ratio (aHR) of 1.785 (95% CI: 1.494–2.134, *P* < 0.0001) compared to those without myocardial injury. The association was even stronger for cardiovascular mortality, with an aHR of 2.155 (95% CI: 1.606–2.893, *P* < 0.0001). When analyzing individual hs-cTn assays, all showed significant associations with mortality outcomes. For all-cause mortality, the Siemens hs-cTn I assay demonstrated the strongest association (aHR: 2.235, 95% CI: 1.642–3.042, *P* < 0.0001), followed by Ortho hs-cTn I (aHR: 1.809, 95% CI: 1.313–2.493, *P* = 0.00029), hs-cTn T (aHR: 1.751, 95% CI: 1.453–2.111, *P* < 0.0001), and Abbott hs-cTn I (aHR: 1.749, 95% CI: 1.180–2.592, *P* = 0.00535). For cardiovascular mortality, all assays showed even higher hazard ratios. The Abbott hs-cTn I assay had the strongest association (aHR: 2.811, 95% CI: 1.579–5.004, *P* = 0.00044), followed closely by Siemens hs-cTn I (aHR: 2.557, 95% CI: 1.552–4.213, *P* = 0.00023), Ortho hs-cTn I (aHR: 2.409, 95% CI: 1.485–3.909, *P* = 0.00037), and hs-cTn T (aHR: 2.230, 95% CI: 1.645–3.023, *P* < 0.0001).

**Table 2:** Adjusted hazard ratios for myocardial injury in non-alcoholic fatty liver disease patients, categorized by high-sensitivity cardiac troponin assay type (Cox proportional hazards regression model).


OUTCOME	hs-cTn ASSAY	aHR (95% CI)*	P-VALUE

All-cause mortality			

	Any hs-cTn assay	1.785 (1.494–2.134)	<0.00001

	hs-cTn T	1.751 (1.453–2.111)	<0.00001

	hs-cTn I Abbott	1.749 (1.180–2.592)	0.00535

	hs-cTn I Ortho	1.809 (1.313–2.493)	0.00029

	hs-cTn I Siemens	2.235 (1.642–3.042)	<0.00001

Cardiovascular mortality			

	Any hs-cTn assay	2.155 (1.606–2.893)	<0.00001

	hs-cTn T	2.230 (1.645–3.023)	<0.00001

	hs-cTn I Abbott	2.811 (1.579–5.004)	0.00044

	hs-cTn I Ortho	2.409 (1.485–3.909)	0.00037

	hs-cTn I Siemens	2.557 (1.552–4.213)	0.00023


The following variables were adjusted: age, sex, race/ethnicity, BMI, education level, marital status, history of malignancy, cardiovascular disease, diabetes mellitus, hypertension, smoking history, statins use, antihypertensive drugs, antidiabetic drugs, antiplatelet drugs, glucose, total cholesterol, diastolic blood pressure, systolic blood pressure, HbA1c (%), ALT; HDL-C; C reactive protein, blood urea nitrogen, uric acid, and sarcopenia; aHR, adjusted hazard ratio. *Reference group: non-alcoholic fatty liver disease patients without myocardial injury.

### 3.3 Sensitivity analysis

**Figure 2:**
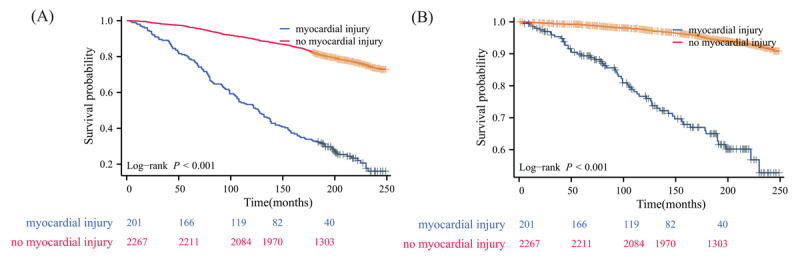
Kaplan–Meier survival curve for patients with non-alcoholic fatty liver disease without known cardiovascular disease using any high-sensitivity cardiac troponin assay to define myocardial injury. (A) all-cause mortality; (B) cardiovascular disease mortality.

To assess the robustness of our findings, we conducted a sensitivity analysis excluding patients with known cardiovascular disease. Among the 36,402,896 weighted NAFLD patients without pre-existing CVD, 5.003% (1,820,145 weighted, 199 unweighted) had myocardial injury. The baseline characteristics of this subgroup were generally similar to the overall cohort (Table S3). Even in this population without known CVD, myocardial injury remained significantly associated with worse clinical outcomes (Figure 2). Furthermore, we analyzed the cumulative survival rates for this subgroup without known CVD. The results, as shown in Table S4, were consistent with our main findings. NAFLD patients with myocardial injury had lower survival rates at all time points compared to those without myocardial injury. The 15-year survival rates were 43.521% for those with myocardial injury versus 86.275% for those without. When examining individual hs-cTn assays, all showed similar patterns of lower survival rates for patients with myocardial injury. The hs-cTn T assay showed the most pronounced difference at 15 years (40.702% vs. 86.083%), while the Abbott hs-cTn I assay showed the smallest difference (53.486% vs. 84.323%). The survival outcomes categorized by the type of hs-cTn assay used to define myocardial injury in NAFLD individuals without CVD are presented in Table S4 and illustrated in Figures S6–S9.

We further conducted Cox proportional hazards regression analysis on this subgroup of NAFLD patients without known CVD (Table 3). After adjusting for baseline characteristics and comorbidities, myocardial injury remained significantly associated with increased risk of all-cause mortality (aHR: 1.771, 95% CI: 1.423–2.204, *P* < 0.0001) and cardiovascular mortality (aHR: 2.203, 95% CI: 1.517–3.198, *P* = 0.00003). Among individual assays, the Siemens hs-cTn I assay showed the strongest association with all-cause mortality (aHR: 2.210, 95% CI: 1.473–3.316, *P* = 0.00013), while the hs-cTn T assay demonstrated the most robust association with cardiovascular mortality (aHR: 2.271, 95% CI: 1.547–3.335, *P* = 0.00003). We further conducted an additional sensitivity analysis excluding participants who died within the first two years of follow-up to address potential reverse causation (Table 4). This approach helps minimize bias from undiagnosed baseline conditions that might have influenced both elevated hs-cTn levels and early mortality, thus providing a more robust assessment of the relationship between hs-cTn levels and long-term mortality outcomes. The results remained consistent with our main findings, with myocardial injury significantly associated with increased risk of all-cause mortality (aHR: 1.746, 95% CI: 1.450–2.101, *P* < 0.0001) and cardiovascular mortality (aHR: 2.092, 95% CI: 1.540–2.843, *P* < 0.0001) when identified by any hs-cTn assay. Finally, we conducted a sensitivity analysis using an alternative definition of hepatic steatosis with a FLI cut-off value of 60 (Table S5). The results remained consistent with our main findings, showing significant associations between myocardial injury and both all-cause and cardiovascular mortality across all hs-cTn assays. This further supports the robustness of our findings, demonstrating that the association between myocardial injury and mortality in NAFLD patients is not dependent on the specific definition of hepatic steatosis used.

**Table 3:** Adjusted hazard ratios for myocardial injury in non-alcoholic fatty liver disease patients without known cardiovascular disease, categorized by high-sensitivity cardiac troponin assay type (Cox proportional hazards regression model).


OUTCOME	hs-cTn ASSAY	aHR (95%CI)	P-VALUE

All-cause mortality			

	Any hs-cTn assay	1.771 (1.423, 2.204)	<0.00001

	hs-cTn T	1.706 (1.354, 2.149)	<0.00001

	hs-cTn I Abbott	1.750 (1.033, 2.976)	0.03759

	hs-cTn I Ortho	1.619 (1.025, 2.557)	0.03870

	hs-cTn I Siemens	2.210 (1.473, 3.316)	0.00013

Cardiovascular mortality			

	Any hs-cTn assay	2.203 (1.517, 3.198)	0.00003

	hs-cTn T	2.271 (1.547, 3.335)	0.00003

	hs-cTn I Abbott	1.771 (0.682, 4.601)	0.24071

	hs-cTn I Ortho	2.142 (1.028, 4.463)	0.04195

	hs-cTn I Siemens	2.639 (1.285, 5.420)	0.00821


The following variables were adjusted: age, sex, race/ethnicity, BMI, education level, marital status, history of malignancy, diabetes mellitus, hypertension, smoking history, statins use, antihypertensive drugs, antidiabetic drugs, antiplatelet drugs, glucose, total cholesterol, diastolic blood pressure, systolic blood pressure, HbA1c (%), ALT; HDL-C; C reactive protein, blood urea nitrogen, uric acid, and sarcopenia; aHR, adjusted hazard ratio. *Reference group: non-alcoholic fatty liver disease patients without myocardial injury.

**Table 4:** Adjusted hazard ratios for myocardial injury in non-alcoholic fatty liver disease patients after excluding participants who died within the first two years of follow-up, categorized by high-sensitivity cardiac troponin assay type (Cox proportional hazards regression model).


OUTCOME	hs-cTn ASSAY	aHR (95%CI)	P-VALUE

All-cause mortality			

	Any hs-cTn assay	1.746 (1.450, 2.101)	<0.00001

	hs-cTn T	1.679 (1.381, 2.042)	<0.00001

	hs-cTn I Abbott	1.724 (1.132, 2.627)	0.01120

	hs-cTn I Ortho	1.947 (1.392, 2.722)	0.00010

	hs-cTn I Siemens	2.173 (1.563, 3.021)	<0.00001

Cardiovascular mortality			

	Any hs-cTn assay	2.092 (1.540, 2.843)	<0.00001

	hs-cTn T	2.147 (1.562, 2.951)	<0.00001

	hs-cTn I Abbott	2.529 (1.340, 4.773)	0.00418

	hs-cTn I Ortho	2.257 (1.332, 3.822)	0.00247

	hs-cTn I Siemens	2.329 (1.354, 4.009)	0.00227


The following variables were adjusted: age, sex, race/ethnicity, BMI, education level, marital status, history of malignancy, diabetes mellitus, hypertension, smoking history, statins use, antihypertensive drugs, antidiabetic drugs, antiplatelet drugs, glucose, total cholesterol, diastolic blood pressure, systolic blood pressure, HbA1c (%), ALT; HDL-C; C reactive protein, blood urea nitrogen, uric acid, and sarcopenia; aHR, adjusted hazard ratio. *Reference group: non-alcoholic fatty liver disease patients without myocardial injury.

We also conducted subgroup analyses to evaluate the consistency of our findings across different population characteristics (Table S6). The association between myocardial injury and mortality remained significant across most subgroups, including sex, age, race/ethnicity, education level, and comorbidities. For all-cause mortality, the association was particularly strong among individuals aged <60 years (aHR: 3.012, 95% CI: 1.626–5.581, *P* = 0.0005 for any hs-cTn assay) and those without hypertension (aHR: 3.407, 95% CI: 2.372–4.892, *P* < 0.0001). Similar patterns were observed for cardiovascular mortality. These consistent results across various sensitivity analyses and subgroup analyses support the robustness of the association between myocardial injury and mortality risk in NAFLD patients.

## 4. Discussion

This study investigated the association between elevated hs-cTn levels and mortality risks in subjects at risk of NAFLD. Our findings reveal a significant association between myocardial injury, as indicated by elevated hs-cTn levels, and increased risks of both all-cause and cardiovascular mortality in subjects at risk of NAFLD. This association remained robust even after adjusting for various demographic, lifestyle, and health-related factors, highlighting the potential of hs-cTn as an important biomarker for predicting adverse outcomes in this population. While NAFLD patients with elevated hs-cTn levels demonstrated higher baseline cardiovascular risk factors, our findings suggest that hs-cTn measurement provides prognostic information beyond traditional risk factors. The persistent association between hs-cTn and mortality after multivariate adjustment, particularly in patients without known cardiovascular disease, indicates that hs-cTn testing could help identify high-risk individuals who might benefit from more intensive preventive interventions, even when traditional risk factors suggest moderate risk. This may be especially valuable for risk stratification in apparently healthy NAFLD patients who have not yet developed overt cardiovascular disease.

To the best of our knowledge, this study provides several novel insights beyond existing literature regarding the prognostic value of hs-cTn in NAFLD patients. Our comprehensive evaluation of four different hs-cTn assays revealed varying strengths of association with mortality outcomes, with the Siemens hs-cTn I assay showing particularly strong predictive value for all-cause mortality (aHR: 2.235, 95% CI: 1.642–3.042) in NAFLD patients. This finding extends beyond previous studies that typically examined fewer assays (19). Furthermore, our detailed analysis of assay-specific thresholds provides practical guidance for clinical risk stratification in NAFLD patients. Importantly, our sensitivity analyses in patients without pre-existing CVD demonstrated that the prognostic value of hs-cTn persists in this subgroup, suggesting its potential utility as a screening tool in apparently healthy NAFLD patients.

The higher prevalence of myocardial injury in NAFLD patients, observed at 7.01% in this study, is a noteworthy finding. NAFLD is often linked to metabolic syndrome (20), which includes conditions such as obesity, insulin resistance, hypertension, and dyslipidemia—all of which are established risk factors for cardiovascular disease (21). The elevated prevalence of myocardial injury in NAFLD patients may reflect the cumulative burden of these cardiovascular risk factors, contributing to subclinical myocardial damage (22,23). The significantly lower survival rates observed for NAFLD patients with myocardial injury indicate a substantial effect of subclinical myocardial damage on long-term outcomes in this population. Patients with elevated hs-cTn levels exhibited markedly worse survival over five, 10, and 15 years compared to those without such injury. This suggests that even in the absence of clinically apparent CVD, myocardial injury in NAFLD patients is a strong predictor of mortality. Importantly, our findings remained robust when analyzing the subgroup of NAFLD patients without known CVD at baseline. In this population, myocardial injury was still significantly associated with increased risk of all-cause mortality (aHR: 1.771, 95% CI: 1.423–2.204) and cardiovascular mortality (aHR: 2.203, 95% CI: 1.517–3.198). These results are particularly noteworthy as they suggest that elevated hs-cTn levels may identify NAFLD patients at increased mortality risk even before clinical manifestations of CVD become apparent.

The analysis of different hs-cTn assays in this study revealed varying strengths of association with mortality outcomes in NAFLD patients, with the Siemens hs-cTn I assay showing the strongest relationship. For all-cause mortality, the Siemens hs-cTn I assay demonstrated the highest adjusted HR of 2.195, indicating that patients with elevated levels detected by this assay had more than twice the risk of death compared to those without myocardial injury. This strong association was consistent across other mortality outcomes as well, suggesting that the Siemens hs-cTn I assay showed superior performance in identifying individuals at higher risk. In comparison, the Roche hs-cTn T assay, while also showing significant associations, demonstrated a slightly lower aHR of 1.800 for all-cause mortality. The other hs-cTn I assays (Ortho and Abbott) also showed significant associations with mortality but with somewhat lower aHRs (Ortho: 1.773, Abbott: 1.604). For cardiovascular mortality, the Siemens hs-cTn I assay showed a strong association with an aHR of 2.543, second only to the Abbott hs-cTn I assay, which had an aHR of 2.664. These findings suggest that while all hs-cTn assays demonstrated significant associations with adverse outcomes, the Siemens hs-cTn I assay showed superior strength of association, making it particularly valuable in clinical settings where risk stratification is crucial. The variability in the strength of associations among the assays highlights the importance of choosing the most appropriate assay for clinical use and the need for assay-specific reference values and interpretation guidelines in practice.

The varying performance among different hs-cTn assays in predicting mortality outcomes warrants further discussion. The superior performance of the Siemens hs-cTn I assay might be partially explained by differences in analytical sensitivities and sex-specific thresholds among the assays. For example, the Siemens assay’s higher thresholds (58 ng/L for males and 39.6 ng/L for females) compared to other assays’ thresholds (e.g., Ortho: 12 ng/L for males and 9 ng/L for females) may result in more specific identification of clinically significant myocardial injury. This higher threshold could potentially reduce false-positive classifications while maintaining high specificity for identifying patients at genuine risk. Conversely, assays with lower thresholds might be more sensitive in detecting minor myocardial injury but could potentially include some cases where the prognostic significance is less clear. These analytical differences among assays should be considered when interpreting our findings and when selecting appropriate assays for clinical use in NAFLD patients.

Our findings align with and extend previous research on cardiac biomarkers in NAFLD and other populations. Liu et al. investigated the association between high-sensitivity cardiac troponin (hs-cTn) and mortality risk in a non-diabetic population using NHANES data. They found that elevated hs-cTnI and hs-cTnT levels were associated with increased risks of all-cause and cardiovascular mortality, with HRs of 2.07 for all-cause mortality and 2.92 for cardiovascular mortality in the case of hs-cTnT (24). However, the HRs observed in our study were higher, particularly for cardiovascular mortality, suggesting that NAFLD patients might face greater risks when elevated hs-cTn levels are present compared to the general non-diabetic population. Moreover, our study extends these findings by focusing specifically on NAFLD patients and by comparing multiple hs-cTn assays. We found that the Siemens hs-cTnI assay showed the strongest association with all-cause mortality in our NAFLD cohort, which provides additional insights into the relative performance of different hs-cTn assays in this specific population. Furthermore, our study reinforces the importance of assessing cardiovascular risk in NAFLD patients, even in the absence of traditional risk factors like diabetes. The study by McEvoy et al. investigated the associations between multiple hs-cTn assays and mortality in a general population without known CVD (8). They reported that each hs-troponin assay was independently associated with all-cause mortality, with hazard ratios ranging from 1.10 to 1.31 per 1 standard deviation increase in log-transformed hs-cTn levels. Similarly, our study in NAFLD patients revealed significant associations between elevated hs-cTn and mortality outcomes. However, the magnitude of association in our NAFLD cohort appears to be stronger. This difference in effect size suggests that elevated hs-cTn levels may carry even greater prognostic significance in NAFLD patients compared to the general population. The stronger associations observed in our cohort emphasize the particular relevance of hs-cTn testing in NAFLD. Bashar et al (18). reported that cancer patients with myocardial injury, defined by elevated hs-cTn, had an adjusted HR of 2.10 (95% CI 2.09–2.10) for all-cause mortality and 2.23 (95% CI 2.22–2.24) for cardiovascular mortality. Similarly, our study in NAFLD patients revealed significant associations between elevated hs-cTn and mortality outcomes. However, the strength of the association in our NAFLD cohort seems to be somewhat lower, which may reflect the differences in the pathophysiological mechanisms between cancer and NAFLD. While both studies compared multiple hs-cTn assays, Bashar et al. found that the Ortho hs-cTnI assay demonstrated the strongest associations with mortality outcomes. In contrast, our study found varying performances among different assays in NAFLD patients. This difference highlights the potential need for assay-specific considerations when interpreting hs-cTn results in different patient populations. These results collectively support the potential use of hs-cTn as a valuable tool for cardiovascular risk stratification across various populations, with potentially enhanced utility in NAFLD patients.

The strong association between elevated hs-cTn levels and increased mortality in NAFLD patients observed in our study can be explained by several potential pathophysiological mechanisms. First, NAFLD is often associated with metabolic syndrome, which includes obesity, insulin resistance, dyslipidemia, and hypertension. These metabolic disturbances can lead to endothelial dysfunction, a precursor to atherosclerosis, thus increasing the risk of myocardial infarction and stroke. Studies have shown that patients with NAFLD have a higher incidence of CVD events, with a pooled odds ratio of 1.6 for myocardial infarction and stroke, indicating a significant association between NAFLD and these cardiovascular events (25). Second, the progression of NAFLD to non-alcoholic steatohepatitis (NASH) is characterized by increased inflammation and fibrosis in the liver. This inflammatory state can contribute to systemic inflammation, which is known to play a crucial role in the development of atherosclerosis and subsequent cardiovascular events. Elevated levels of inflammatory markers such as C-reactive protein (CRP) and cytokines may further exacerbate cardiovascular risk in NAFLD patients (26). Additionally, NAFLD is associated with an atherogenic lipid profile, characterized by high triglycerides, low HDL cholesterol, and increased small, dense LDL particles. This dyslipidemia promotes the development of atherosclerosis, which can lead to coronary artery disease and subsequent myocardial injury (27,28). Third, NAFLD is also associated with increased oxidative stress, both in the liver and systemically. Oxidative stress can lead to lipid peroxidation, protein oxidation, and DNA damage in various tissues, including the myocardium. This oxidative damage can contribute to subtle, chronic myocardial injury (29,30,31). Furthermore, NAFLD has been associated with impaired endothelial function, which is a key early step in the development of atherosclerosis. Endothelial dysfunction can lead to reduced coronary flow reserve and impaired myocardial perfusion, potentially causing subclinical myocardial injury (32). Furthermore, it’s important to note that NAFLD and cardiovascular disease share several risk factors, which may contribute to the observed association between myocardial injury and mortality in NAFLD patients. Obesity is a primary risk factor for both NAFLD and CVD. It contributes to insulin resistance, inflammation, and dyslipidemia, which are central to the pathogenesis of both conditions (1). Insulin resistance is a key pathophysiological feature of NAFLD and a major risk factor for CVD. It promotes hepatic fat accumulation and contributes to atherogenesis (33). Moreover, NAFLD is considered the hepatic manifestation of metabolic syndrome. The components of metabolic syndrome (central obesity, insulin resistance, hypertension, and dyslipidemia) are shared risk factors for both NAFLD and CVD (34,35).

The association between NAFLD and increased cardiovascular mortality likely involves multiple complex pathways beyond shared risk factors like insulin resistance and dyslipidemia (4). NAFLD may directly contribute to cardiovascular risk through several mechanisms. Hepatic inflammation in NAFLD can lead to increased production of pro-inflammatory cytokines and procoagulant factors, promoting systemic inflammation and a prothrombotic state (3,36). The altered lipid metabolism in NAFLD, characterized by increased very-low-density lipoprotein production and decreased high-density lipoprotein levels, may accelerate atherosclerosis (37). Furthermore, NAFLD-associated hepatic insulin resistance can exacerbate whole-body insulin resistance, potentially leading to endothelial dysfunction and vascular inflammation (38,39). Emerging evidence also suggests that hepatokines produced by the fatty liver, such as fetuin-A and fibroblast growth factor 21, may directly influence cardiac function and metabolism (40,41). These multifaceted interactions underscore the complex relationship between NAFLD and cardiovascular health, highlighting the need for comprehensive cardiovascular risk assessment in NAFLD patients (42).

However, our study has several important limitations. First, the observational design prevents establishing definitive causal links between elevated hs-cTn levels and mortality outcomes, and residual confounding cannot be ruled out despite comprehensive adjustment. Second, the diagnosis of NAFLD was based on FLI rather than liver biopsy or imaging techniques, which may have led to some misclassification. A recent meta-analysis has highlighted limitations in FLI’s discriminatory performance (43), particularly for values between 30–60, suggesting subjects identified through this index should be considered ‘at risk of NAFLD’ rather than definitive cases. Third, our sensitivity analysis in patients without known CVD, while supporting our main findings, was limited by a relatively small sample size. Nevertheless, the consistency of results across different sensitivity analyses and the stability of effect estimates after sequential adjustment support the robustness of our findings. Fourth, the single time-point measurement of hs-cTn levels may not capture the dynamic nature of troponin release. Finally, our study lacks echocardiographic data, which could provide valuable insights into cardiac structure and function, including assessment of heart failure, valvular diseases, and pulmonary hypertension that might contribute to poor prognosis in this population. Future studies incorporating echocardiographic examinations alongside hs-troponin measurements could offer more comprehensive cardiovascular risk assessment in individuals at risk of NAFLD.

## 5. Conclusion

This large, nationally representative study demonstrates that myocardial injury, defined by elevated hs-cTn levels, is independently associated with increased all-cause and cardiovascular mortality risks in the population at risk of NAFLD. This association persisted after adjusting for various factors and in patients without pre-existing cardiovascular disease. The Siemens hs-cTn I assay demonstrated the strongest association with all-cause mortality. These findings highlight the potential of hs-cTn as a valuable prognostic marker in the population at risk of NAFLD, even in those without clinically apparent cardiovascular disease. Routine hs-cTn assessment could aid in risk stratification and guide targeted preventive strategies. Future research should evaluate the impact of hs-cTn-guided interventions on clinical outcomes in NAFLD patients.

## Data Accessibility Statement

The data utilized in this study were obtained from publicly accessible resources, specifically from the NHANES database, which can be accessed at https://wwwn.cdc.gov/nchs/nhanes/default.aspx.

## Additional File

The additional file for this article can be found as follows:

10.5334/gh.1427.s1 Supplementary Material. Supplementary Tables S1–S6 and Supplementary Figures S1–S9.
