# Supplementary material for: Association Between High Sensitivity Cardiac Troponin and All-Cause and Cardiovascular Mortality in Adults at Risk of Non-Alcoholic Fatty Liver Disease: A Cohort Study
Source: Glob Heart. 2025 Apr 29;20(1):40. doi: 10.5334/gh.1427 (PMC12047645; doi:10.5334/gh.1427)
Supplement: Supplementary Material. — Supplementary Tables S1–S6 and Supplementary Figures S1–S9. [file gh-20-1-1427-s1.pdf]

Supplementary material

Table of contents

Supplementary Table S1 ……………………………………………………………………… Page 2

Supplementary Table S2 ……………………………………………………………………… Page 3

Supplementary Table S3 ……………………………………………………………………… Page 6

Supplementary Table S4 ……………………………………………………………………… Page 8

Supplementary Table S5 ……………………………………………………………………… Page 9

Supplementary Table S6 ……………………………………………………………………… Page 10

Supplementary Figure S1 …………………………………………………………………….. Page 14

Supplementary Figure S2 …………………………………………………………………….. Page 15

Supplementary Figure S3 …………………………………………………………………….. Page 16

Supplementary Figure S4 …………………………………………………………………….. Page 17

Supplementary Figure S5 …………………………………………………………………….. Page 18

Supplementary Figure S6 …………………………………………………………………….. Page 19

Supplementary Figure S7 ………………………………………………………………….… Page 20

Supplementary Figure S8 ………………………………………………………………….… Page 21

Supplementary Figure S9 ………………………………………………………………….… Page 22

Table S1 The median and interquartile range of troponin values, stratified by the type of hs-cTn (high-sensitivity cardiac troponin) assay used to define myocardial injury.

hs-cTn assay	Without Myocardial Injury	Myocardial Injury
hs-cTn T, median (IQR), ng/L	5.49 (3.92, 8.08)	22.62 (16.48, 29.04)
hs-cTn I Abbott, median (IQR), ng/L	1.80 (1.20, 2.80)	7.30 (3.70, 15.50)
hs-cTn I Siemens, median (IQR), ng/L	2.63 (1.35, 4.55)	11.09 (5.34, 35.76)
hs-cTn I Ortho, median (IQR), ng/L	0.42 (0.14, 0.95)	3.55 (1.28, 8.41)

A total of 41,828,861 weighted records (2,581 unweighted) representing adults with NAFLD were included in this analysis. All participants were at least 18 years old and had complete vital status information. Myocardial injury, defined as an elevation in at least one high-sensitivity cardiac troponin (hs-cTn) assay, was present in 7.01% of the study population (2,932,203 weighted records, 322 unweighted). The study utilized four hs-cTn assays: one for troponin T (Roche) and three for troponin I (Abbott, Siemens, and Ortho). Over a median follow-up of 200 months, 937 all-cause deaths occurred, including 319 cardiovascular disease-related deaths. The distribution of hs-cTn values in both study arms (with and without myocardial injury) is presented in **Table S1**. Note: For continuous variables: survey-weighted Median (Q1, Q3), P-value was by survey-weighted linear regression (svyglm); hs-cTn: high-sensitivity cardiac troponin; IQR: Interquartile Range.

Table S2. Baseline characteristics of non-alcoholic fatty liver disease categorized by myocardial injury status

	Without myocardial injury (n=38896658, 92.991%)	Myocardial injury (n=2932203, 7.009%)	P-value
Unweighted records, n	2529	322	
Age (years)	47.000 (36.000 ,60.000)	71.000 (62.000 ,81.000)	<0.0001
Family income-poverty ratio	3.230 (1.630 ,5.000)	2.310 (1.300 ,4.130)	0.0005
BMI (kg/m^2)	29.890 (27.300 ,33.610)	29.320 (26.390 ,34.490)	0.3956
Waist circumference (cm)	102.400 (96.000 ,111.000)	104.600 (96.700 ,114.100)	0.1281
Diastolic blood pressure (mmHg)	74.000 (67.000 ,81.000)	67.000 (60.000 ,76.000)	<0.0001
Systolic blood pressure (mmHg)	122.000 (113.000 ,134.000)	132.000 (115.000 ,152.000)	<0.0001
Glucose (mg/dl)	98.400 (91.600 ,107.100)	105.300 (94.800 ,124.600)	0.0001
HbA1c (%)	5.400 (5.200 ,5.700)	5.600 (5.300 ,6.100)	<0.0001
ALT (IU/L)	24.000 (18.000 ,32.000)	20.000 (16.000 ,26.000)	0.0017
AST (IU/L)	22.000 (19.000 ,27.000)	23.000 (19.000 ,28.000)	0.9911
Creatinine (mg/dl)	0.800 (0.700 ,1.000)	1.000 (0.800 ,1.300)	<0.0001
Uric acid (mg/dl)	5.700 (4.800 ,6.600)	6.200 (5.300 ,7.500)	<0.0001
Blood urea nitrogen (mg/dl)	13.000 (11.000 ,16.000)	18.000 (14.000 ,23.000)	<0.0001
Triglyceride (mg/dl)	144.000 (103.000 ,201.000)	149.000 (107.000 ,211.000)	0.6956
Total cholesterol (mg/dl)	203.000 (179.000 ,231.000)	203.000 (176.000 ,230.000)	0.5026
HDL cholesterol (mg/dl)	45.000 (38.000 ,54.000)	46.000 (38.000 ,56.000)	0.0581
LDL cholesterol (mg/dl)	124.000 (102.000 ,148.000)	121.000 (96.000 ,145.000)	0.198
C reactive protein (mg/dl)	0.270 (0.120 ,0.540)	0.340 (0.170 ,0.680)	0.0235
eGFR (mL/min/1.73m2)	93.159 (79.810 ,106.461)	61.873 (45.891 ,80.772)	<0.0001

Sex			0.0123
Female	44.830 (42.534 ,47.150)	53.619 (46.732 ,60.371)	
Male	55.170 (52.850 ,57.466)	46.381 (39.629 ,53.268)	
Race/Ethnicity			0.0616
Mexican American	7.033 (5.230 ,9.395)	5.421 (3.004 ,9.591)	
Non-Hispanic Black	11.221 (8.901 ,14.053)	14.965 (10.220 ,21.389)	
Non-Hispanic White	72.345 (67.554 ,76.672)	74.648 (67.852 ,80.422)	
Other	9.401 (6.672 ,13.090)	4.965 (2.275 ,10.496)	
Education level			<0.0001
Less than high school	20.756 (18.513 ,23.193)	30.273 (24.991 ,36.133)	
High school graduates	27.854 (25.531 ,30.303)	32.297 (25.976 ,39.338)	
Above high school	51.390 (48.259 ,54.510)	37.430 (31.003 ,44.334)	
Marital status			<0.0001
Unmarried	29.159 (26.675 ,31.774)	45.027 (37.590 ,52.694)	
Married	70.841 (68.226 ,73.325)	54.973 (47.306 ,62.410)	
Malignancy			<0.0001
No	92.243 (90.594 ,93.623)	80.964 (74.022 ,86.392)	
Yes	7.757 (6.377 ,9.406)	19.036 (13.608 ,25.978)	
CVD			<0.0001
No	91.318 (89.709 ,92.695)	62.362 (55.605 ,68.671)	
Yes	8.682 (7.305 ,10.291)	37.638 (31.329 ,44.395)	
Smoking status			0.0046
Never	51.596 (47.993 ,55.183)	44.576 (38.726 ,50.580)	
Former	30.051 (26.984 ,33.308)	41.991 (34.192 ,50.212)	
Now	18.353 (16.565 ,20.286)	13.433 (8.444 ,20.705)	
Diabetes			<0.0001
No	89.060 (87.565 ,90.394)	69.754 (60.240 ,77.830)	
Yes	10.940 (9.606 ,12.435)	30.246 (22.170 ,39.760)	

Hypertension			<0.0001
No	56.457 (53.253 ,59.608)	29.770 (21.904 ,39.048)	
Yes	43.543 (40.392 ,46.747)	70.230 (60.952 ,78.096)	
Alcohol drinker			0.7464
No	43.994 (38.971 ,49.143)	42.174 (33.340 ,51.539)	
Yes	56.006 (50.857 ,61.029)	57.826 (48.461 ,66.660)	
Statins			<0.0001
No	87.277 (85.616 ,88.772)	70.657 (62.977 ,77.318)	
Yes	12.723 (11.228 ,14.384)	29.343 (22.682 ,37.023)	
Antihypertensive drugs			<0.0001
No	80.615 (77.770 ,83.175)	56.898 (50.273 ,63.286)	
Yes	19.385 (16.825 ,22.230)	43.102 (36.714 ,49.727)	
Antidiabetic drugs			<0.0001
No	93.308 (92.114 ,94.333)	79.202 (73.774 ,83.755)	
Yes	6.692 (5.667 ,7.886)	20.798 (16.245 ,26.226)	
Antiplatelet drugs			<0.0001
No	98.562 (97.793 ,99.066)	94.015 (90.037 ,96.467)	
Yes	1.438 (0.934 ,2.207)	5.985 (3.533 ,9.963)	
Sarcopenia			<0.0001
No	88.881 (86.357 ,90.988)	71.159 (61.689 ,79.082)	
Yes	11.119 (9.012 ,13.643)	28.841 (20.918 ,38.311)	

NAFLD patients with myocardial injury were older (71 vs. 47 years, P < 0.001) and more likely to be female (53.619% vs. 44.830%, P = 0.0123). While there was no significant difference in race/ethnicity distribution (P = 0.0616), those with myocardial injury had a higher prevalence of comorbidities and cardiovascular risk factors. These included cardiovascular disease (37.638% vs. 8.682%, P < 0.0001), diabetes mellitus (30.246% vs. 10.940%, P < 0.0001), hypertension (70.230% vs. 43.543%, P < 0.0001), malignancy (19.036% vs. 7.757%, P < 0.0001),

and sarcopenia (28.841% vs. 11.119%, P < 0.0001). Patients with myocardial injury also had higher levels of glucose, HbA1c, and creatinine (all P < 0.001). They were more likely to use statins (29.343% vs. 12.723%, P < 0.0001), antihypertensive drugs (43.102% vs. 19.385%, P < 0.0001), and antidiabetic drugs (20.798% vs. 6.692%, P < 0.0001). Interestingly, there were no significant differences in BMI, waist circumference, or alcohol consumption between the two groups. **Table S2** presents the baseline characteristics of the study population stratified by myocardial injury status. For continuous variables: survey-weighted Median (Q1, Q3), P-value was by survey-weighted linear regression (svyglm); For categorical variables: survey-weighted percentage (95% CI), P-value was by survey-weighted Chi-square test (svytable).

Table S3 Baseline characteristics of non-alcoholic fatty liver disease patients without known cardiovascular disease

	Without myocardial injury (n=34582751, 95.00%)	Myocardial injury (n=1820145, 5.00%)	P-value
Unweighted records, n	2092	199	
Age (years)	47.00 (36.00,59.00)	69.00 (57.00,80.00)	<0.0001
Family income-poverty ratio	3.32 (1.70,5.00)	2.46 (1.50,3.72)	0.0027
BMI (kg/m2)	29.89 (27.30,33.58)	29.20 (26.31,33.94)	0.3721
Waist circumference (cm)	102.20 (96.00,110.80)	104.70 (96.40,112.30)	0.3986
Diastolic blood pressure (mmHg)	75.00 (68.00,82.00)	68.00 (61.00,75.00)	<0.0001
Systolic blood pressure (mmHg)	122.00 (113.00,134.00)	130.00 (112.00,147.00)	0.0004
Glucose (mg/dl)	97.90 (91.40,106.10)	105.20 (94.30,120.80)	0.0011
HbA1c (%)	5.40 (5.20,5.60)	5.50 (5.30,6.00)	0.0015
ALT (IU/L)	24.00 (18.00,32.00)	20.00 (16.00,28.00)	0.0562
AST (IU/L)	22.00 (19.00,27.00)	24.00 (19.00,28.00)	0.3693
Creatinine (mg/dl)	0.80 (0.70,1.00)	0.90 (0.70,1.10)	0.0001
Uric acid (mg/dl)	5.70 (4.80,6.60)	6.00 (5.20,7.10)	0.0022
Blood urea nitrogen (mg/dl)	13.00 (11.00 ,16.00)	16.00 (12.00 ,21.00)	<0.0001
Triglyceride (mg/dl)	143.00 (102.00,199.00)	153.00 (104.00,212.00)	0.8153
Total cholesterol (mg/dl)	204.00 (180.00232.00)	206.00 (177.00,230.00)	0.891
HDL cholesterol (mg/dl)	45.00 (39.00,54.00)	46.00 (37.00,56.00)	0.1185
LDL cholesterol (mg/dl)	126.00 (103.00 ,149.00)	128.00 (98.00,146.00)	0.6983
C reactive protein (mg/dl)	0.27 (0.12,0.54)	0.34 (0.13,0.68)	0.0515
eGFR (mL/min/1.73m2)	93.73 (80.87,106.46)	71.50 (51.97,91.86)	<0.0001
Sex			0.0156
Female	45.68 (43.11 ,48.27)	55.49 (47.52 ,63.19)	
Male	54.32 (51.73 ,56.89)	44.51 (36.81 ,52.48)	
Race/Ethnicity			0.0278
Mexican American	7.28 (5.45 ,9.66)	6.31 (3.44 ,11.28)	
Non-Hispanic Black	11.35 (8.99 ,14.23)	14.03 (8.87 ,21.48)	
Non-Hispanic White	72.00 (67.03 ,76.48)	76.92 (67.75 ,84.10)	

Other	9.37 (6.48 ,13.37)	2.74 (0.84 ,8.61)	
Education level			0.0005
Less than high school	19.76 (17.40 ,22.35)	27.82 (22.17 ,34.28)	
High school graduates	27.92 (25.57 ,30.40)	36.11 (28.74 ,44.21)	
Above high school	52.32 (49.04 ,55.59)	36.06 (27.66 ,45.42)	
Marital status			<0.0001
Unmarried	27.40 (24.88 ,30.08)	48.87 (39.92 ,57.90)	
Married	72.60 (69.92 ,75.12)	51.13 (42.10 ,60.08)	
Malignancy			<0.0001
No	93.05 (91.48 ,94.34)	80.39 (72.23 ,86.59)	
Yes	6.95 (5.66 ,8.52)	19.61 (13.41 ,27.77)	
Smoking status			0.0424
Never	53.21 (49.47 ,56.91)	43.38 (34.72 ,52.46)	
Former	28.46 (25.32 ,31.81)	39.16 (30.73 ,48.30)	
Now	18.34 (16.18 ,20.72)	17.46 (10.55 ,27.49)	
Diabetes			<0.0001
No	90.22 (88.47 ,91.74)	75.87 (66.32 ,83.40)	
Yes	9.78 (8.26 ,11.53)	24.13 (16.60 ,33.68)	
Hypertension			<0.0001
No	57.70 (54.54 ,60.79)	35.19 (26.30 ,45.24)	
Yes	42.30 (39.21 ,45.46)	64.81 (54.76 ,73.70)	
Alcohol drinker			0.5768
No	44.37 (39.04 ,49.82)	40.06 (26.89 ,54.84)	
Yes	55.63 (50.18 ,60.96)	59.94 (45.16 ,73.11)	
Statins			0.0007
No	89.73 (88.20 ,91.08)	77.38 (66.59 ,85.44)	
Yes	10.27 (8.92 ,11.80)	22.62 (14.56 ,33.41)	
Antihypertensive drugs			<0.0001
No	82.66 (79.84 ,85.16)	65.27 (56.93 ,72.76)	
Yes	17.34 (14.84 ,20.16)	34.73 (27.24 ,43.07)	
Antidiabetic drugs			0.0001
No	93.78 (92.47 ,94.88)	83.55 (75.50 ,89.33)	
Yes	6.22 (5.12 ,7.53)	16.45 (10.67 ,24.50)	
Antiplatelet drugs			0.8264
No	99.61 (99.21 ,99.81)	99.52 (97.37 ,99.91)	
Yes	0.39 (0.19 ,0.79)	0.48 (0.09 ,2.63)	

For continuous variables: survey-weighted Median (Q1, Q3), P-value was by survey-weighted linear regression (svyglm); For categorical variables: survey-weighted percentage (95% CI), P-value was by survey-weighted Chi-square test (svytable)

Table S4 Cumulative survival rates at 1, 5, 10, and 15 years for non-alcoholic fatty liver disease patients without known cardiovascular disease, categorized by the specific high-sensitivity cardiac troponin test used to identify myocardial injury (unadjusted)

hs-cTn assay/time point*	Without myocardial injury	Myocardial injury
Any hs-cTn assay (weighted)	34581555	1821341
1 year, %	99.74	98.36
5 years, %	97.42	82.27
10 years, %	92.3	60.3
15 years, %	86.28	43.52
hs-cTn T (weighted)	34841194	1561702
1 year, %	99.74	98.08
5 years, %	97.39	80.51
10 years, %	92.21	57.05
15 years, %	86.08	40.7
hs-cTn I Abbott (weighted)	36181900	220996
1 year, %	99.69	96.31
5 years, %	96.71	88.28
10 years, %	90.84	68.33
15 years, %	84.32	53.49
hs-cTn I Siemens (weighted)	36067172	335723
1 year, %	99.69	97.57
5 years, %	96.73	89.27
10 years, %	90.97	61.39
15 years, %	84.52	42.89
hs-cTn I Ortho (weighted)	36135044	267852
1 year, %	99.69	96.95
5 years, %	96.72	88.71
10 years, %	90.94	58.69
15 years, %	84.43	43.94

*All statistical analyses and derived estimates utilize weighted data records

Table S5 Adjusted hazard ratios for myocardial injury in patients with non-alcoholic fatty liver disease, using an alternative definition of hepatic steatosis (FLI cut-off value of 60), stratified by high-sensitivity cardiac troponin assay type (Cox proportional hazards regression model).

Outcome	hs-cTn assay	aHR (95% CI)*	P-value
All-cause mortality			
	Any hs-cTn assay	1.814 (1.520-2.165)	<0.00001
	hs-cTn T	1.800 (1.496-2.165)	<0.00001
	hs-cTn I Abbott	1.604 (1.091-2.360)	0.01632
	hs-cTn I Ortho	1.773 (1.287-2.442)	0.00046
	hs-cTn I Siemens	2.195 (1.614-2.986)	<0.00001
Cardiovascular mortality			
	Any hs-cTn assay	2.191 (1.637-2.933)	<0.00001
	hs-cTn T	2.256 (1.669-3.049)	<0.00001
	hs-cTn I Abbott	2.664 (1.520-4.666)	0.00062
	hs-cTn I Ortho	2.397 (1.479-3.885)	0.00039
	hs-cTn I Siemens	2.543 (1.548-4.180)	0.00023

The following variables were adjusted: age, sex, race/ethnicity, BMI, education level, marital status, history of malignancy, cardiovascular disease, diabetes mellitus, hypertension, smoking history, statins use, antihypertensive drugs, antidiabetic drugs, antiplatelet drugs, glucose, total cholesterol, diastolic blood pressure, systolic blood pressure, HbA1c (%), ALT; HDL-C; C reactive protein, blood urea nitrogen, and uric acid

aHR, adjusted hazard ratio.

*Reference group: non-alcoholic fatty liver disease patients without myocardial injury.

Table S6 Adjusted hazard ratios for all-cause and cardiovascular disease mortality in non-alcoholic fatty liver disease patients with myocardial injury, stratified by high-sensitivity cardiac troponin assay type and subgroup characteristics

	Any hs-cTn assay	hs-cTn T	hs-cTn I Abbott	hs-cTn I Ortho	hs-cTn I Siemens
All-cause mortality*					
Sex					
Female	1.802 (1.366, 2.377) <0.0001	1.920 (1.444, 2.554) <0.0001	1.069 (0.597, 1.913) 0.8227	1.603 (0.897, 2.865) 0.1113	1.906 (1.172, 3.100) 0.0093
Male	1.852 (1.464, 2.342) <0.0001	1.699 (1.326, 2.178) <0.0001	2.188 (1.290, 3.712) 0.0037	1.823 (1.219, 2.725) 0.0034	2.270 (1.488, 3.463) 0.0001
Age					
<60	3.012 (1.626, 5.581) 0.0005	2.734 (1.319, 5.668) 0.0068	2.703 (0.724, 10.085) 0.1388	2.534 (0.821, 7.824) 0.1060	3.381 (1.185, 9.646) 0.0227
>=60	2.237 (1.864, 2.684) <0.0001	2.277 (1.880, 2.757) <0.0001	1.496 (0.995, 2.248) 0.0528	2.110 (1.512, 2.943) <0.0001	2.041 (1.475, 2.824) <0.0001
Race/Ethnicity					
Mexican American	2.718 (1.782, 4.146) <0.0001	2.669 (1.683, 4.234) <0.0001	1.773 (0.586, 5.367) 0.3109	4.858 (2.229, 10.589) <0.0001	2.245 (1.139, 4.425) 0.0194
Non-Hispanic Black	1.633 (1.016, 2.625) 0.0427	1.545 (0.938, 2.544) 0.0875	1.760 (0.887, 3.493) 0.1060	0.973 (0.496, 1.910) 0.9365	2.624 (1.364, 5.047) 0.0038
Non-Hispanic White	1.651 (1.308, 2.083) <0.0001	1.698 (1.340, 2.152) <0.0001	1.123 (0.603, 2.091) 0.7155	1.477 (0.907, 2.404) 0.1170	2.041 (1.261, 3.306) 0.0037
Education level					
Less than high school	2.185 (1.675, 2.850) <0.0001	2.208 (1.660, 2.937) <0.0001	2.000 (1.163, 3.439) 0.0122	1.903 (1.165, 3.107) 0.0101	2.192 (1.399, 3.433) 0.0006
High school graduates	1.645 (1.096, 2.470) 0.0164	1.578 (1.037, 2.402) 0.0331	1.444 (0.425, 4.910) 0.5558	1.686 (0.879, 3.234) 0.1163	2.604 (1.206, 5.624) 0.0148
Above high school	1.785 (1.287, 2.477) 0.0005	1.748 (1.251, 2.443) 0.0011	1.307 (0.642, 2.660) 0.4603	1.791 (0.956, 3.355) 0.0689	1.785 (0.995, 3.200) 0.0519
Marital status					
Unmarried	1.658 (1.250,	1.712 (1.282,	1.419 (0.746,	1.241 (0.663,	2.110 (1.181,

	2.198) 0.0004	2.286) 0.0003	2.700) 0.2866	2.322) 0.4993	3.770) 0.0117
Married	2.006 (1.590, 2.531) <0.0001	1.918 (1.499, 2.455) <0.0001	1.681 (0.997, 2.836) 0.0515	1.973 (1.332, 2.921) 0.0007	2.284 (1.537, 3.394) <0.0001
Malignancy					
No	1.784 (1.465, 2.174) <0.0001	1.764 (1.433, 2.171) <0.0001	1.366 (0.894, 2.088) 0.1499	1.715 (1.212, 2.425) 0.0023	1.977 (1.402, 2.788) 0.0001
Yes	1.855 (1.159, 2.972) 0.0101	1.847 (1.135, 3.007) 0.0136	2.140 (0.655, 6.987) 0.2077	2.040 (0.779, 5.340) 0.1465	3.106 (1.436, 6.716) 0.0040
CVD					
No	1.831 (1.477, 2.270) <0.0001	1.778 (1.417, 2.229) <0.0001	1.647 (0.973, 2.787) 0.0631	1.545 (0.979, 2.438) 0.0617	2.137 (1.426, 3.202) 0.0002
Yes	1.755 (1.245, 2.474) 0.0013	1.794 (1.262, 2.551) 0.0011	1.784 (0.946, 3.366) 0.0739	2.427 (1.490, 3.951) 0.0004	2.612 (1.521, 4.484) 0.0005
Smoking status					
Never	1.847 (1.413, 2.416) <0.0001	1.797 (1.365, 2.366) <0.0001	1.666 (0.929, 2.988) 0.0866	2.084 (1.330, 3.265) 0.0014	2.681 (1.690, 4.254) <0.0001
Former	1.899 (1.431, 2.519) <0.0001	1.941 (1.447, 2.604) <0.0001	1.573 (0.850, 2.911) 0.1489	1.686 (0.990, 2.871) 0.0544	1.854 (1.081, 3.179) 0.0248
Now	1.850 (1.079, 3.174) 0.0254	1.456 (0.786, 2.697) 0.2318	4.919 (1.270, 19.049) 0.0211	2.033 (0.639, 6.470) 0.2297	3.484 (1.419, 8.550) 0.0064
Diabetes					
No	1.909 (1.544, 2.360) <0.0001	1.800 (1.439, 2.251) <0.0001	1.951 (1.229, 3.100) 0.0046	1.917 (1.309, 2.806) 0.0008	2.364 (1.629, 3.430) <0.0001
Yes	1.607 (1.144, 2.257) 0.0063	1.711 (1.196, 2.448) 0.0033	1.389 (0.626, 3.082) 0.4187	1.612 (0.874, 2.973) 0.1266	1.995 (1.073, 3.711) 0.0290
Hypertension					
No	3.407 (2.372, 4.892) <0.0001	3.036 (2.063, 4.467) <0.0001	2.208 (0.807, 6.044) 0.1230	2.840 (1.208, 6.676) 0.0167	4.319 (2.099, 8.886) <0.0001
Yes	1.627 (1.327, 1.994) <0.0001	1.653 (1.336, 2.045) <0.0001	1.618 (1.050, 2.493) 0.0292	1.800 (1.262, 2.566) 0.0012	1.998 (1.409, 2.835) 0.0001
Cardiovascular					

disease mortality					
Sex					
Female	1.945 (1.208, 3.134) 0.0062	2.035 (1.246, 3.321) 0.0045	1.627 (0.659, 4.018) 0.2914	1.842 (0.700, 4.851) 0.2161	3.006 (1.409, 6.411) 0.0044
Male	2.285 (1.556, 3.355) <0.0001	2.172 (1.463, 3.223) 0.0001	4.070 (1.959, 8.456) 0.0002	3.083 (1.698, 5.597) 0.0002	2.069 (1.011, 4.231) 0.0465
Age					
<60	2.309 (0.699, 7.631) 0.1698	2.687 (0.792, 9.114) 0.1126	4.270 (0.613, 29.714) 0.1425	3.154 (0.519, 19.169) 0.2123	3.463 (0.533, 22.510) 0.1934
>=60	3.000 (2.228, 4.040) <0.0001	3.079 (2.260, 4.197) <0.0001	2.333 (1.275, 4.268) 0.0060	3.078 (1.857, 5.103) <0.0001	2.584 (1.539, 4.337) 0.0003
Race/Ethnicity					
Mexican American	2.093 (0.948, 4.624) 0.0677	3.050 (1.336, 6.964) 0.0081	0.585 (0.042, 8.103) 0.6892	3.324 (0.703, 15.706) 0.1295	1.819 (0.473, 6.993) 0.3837
Non-Hispanic Black	1.653 (0.688, 3.973) 0.2611	1.803 (0.736, 4.414) 0.1971	2.136 (0.684, 6.672) 0.1915	0.693 (0.201, 2.396) 0.5628	2.407 (0.733, 7.907) 0.1478
Non-Hispanic White	2.366 (1.636, 3.423) <0.0001	2.312 (1.588, 3.367) <0.0001	2.961 (1.348, 6.505) 0.0069	3.785 (2.043, 7.013) <0.0001	4.016 (2.024, 7.965) <0.0001
Education level					
Less than high school	2.489 (1.582, 3.917) <0.0001	2.892 (1.799, 4.649) <0.0001	2.359 (0.942, 5.907) 0.0670	1.663 (0.703, 3.934) 0.2474	1.141 (0.459, 2.835) 0.7759
High school graduates	2.352 (1.187, 4.657) 0.0142	2.214 (1.105, 4.439) 0.0251	4.285 (0.805, 22.817) 0.0881	2.326 (0.839, 6.448) 0.1048	5.352 (1.805, 15.871) 0.0025
Above high school	2.031 (1.162, 3.551) 0.0129	1.911 (1.084, 3.367) 0.0251	2.760 (1.106, 6.888) 0.0296	3.630 (1.583, 8.320) 0.0023	2.835 (1.189, 6.758) 0.0187
Marital status					
Unmarried	2.145 (1.332, 3.455) 0.0017	2.178 (1.340, 3.538) 0.0017	2.594 (0.908, 7.415) 0.0752	1.117 (0.352, 3.545) 0.8514	7.078 (3.031, 16.527) <0.0001
Married	2.259 (1.531, 3.334) <0.0001	2.330 (1.560, 3.479) <0.0001	2.805 (1.343, 5.858) 0.0061	2.919 (1.627, 5.237) 0.0003	1.845 (0.921, 3.697) 0.0840
Malignancy					
No	2.039 (1.474,	2.140 (1.530,	2.237 (1.200,	2.067 (1.198,	2.129 (1.213,

	2.820) <0.0001	2.993) <0.0001	4.170) 0.0113	3.566) 0.0091	3.737) 0.0085
Yes	5.042 (1.931, 13.169) 0.0010	4.500 (1.714, 11.816) 0.0023	4.875 (0.751, 31.667) 0.0970	8.047 (1.902, 34.041) 0.0046	7.035 (1.808, 27.377) 0.0049
CVD					
No	2.250 (1.557, 3.252) <0.0001	2.328 (1.593, 3.403) <0.0001	1.709 (0.660, 4.429) 0.2698	2.106 (1.015, 4.370) 0.0456	2.550 (1.245, 5.223) 0.0105
Yes	1.920 (1.152, 3.199) 0.0122	1.955 (1.167, 3.273) 0.0108	4.786 (2.069, 11.072) 0.0003	3.292 (1.622, 6.681) 0.0010	2.834 (1.277, 6.288) 0.0104
Smoking status					
Never	2.133 (1.329, 3.425) 0.0017	2.120 (1.308, 3.436) 0.0023	1.743 (0.638, 4.766) 0.2788	2.063 (0.956, 4.450) 0.0649	3.261 (1.463, 7.269) 0.0038
Former	2.624 (1.691, 4.074) <0.0001	2.596 (1.650, 4.084) <0.0001	3.117 (1.395, 6.967) 0.0056	3.115 (1.514, 6.406) 0.0020	2.626 (1.231, 5.602) 0.0125
Now	1.691 (0.527, 5.423) 0.3767	2.031 (0.607, 6.793) 0.2503	14.158 (1.359, 147.497) 0.0267	3.724 (0.345, 40.239) 0.2790	2.840 (0.330, 24.469) 0.3422
Diabetes					
No	2.601 (1.833, 3.691) <0.0001	2.584 (1.797, 3.718) <0.0001	3.168 (1.605, 6.255) 0.0009	3.450 (2.004, 5.939) <0.0001	2.719 (1.469, 5.034) 0.0015
Yes	1.897 (1.073, 3.352) 0.0276	1.987 (1.103, 3.578) 0.0222	3.019 (0.969, 9.407) 0.0567	1.011 (0.318, 3.216) 0.9853	3.240 (1.234, 8.510) 0.0170
Hypertension					
No	6.245 (3.263, 11.952) <0.0001	5.845 (2.972, 11.498) <0.0001	8.640 (2.073, 36.010) 0.0031	16.669 (4.735, 58.687) <0.0001	4.903 (1.344, 17.886) 0.0160
Yes	1.717 (1.234, 2.389) 0.0013	1.836 (1.306, 2.580) 0.0005	2.447 (1.281, 4.672) 0.0067	1.942 (1.110, 3.398) 0.0201	2.407 (1.384, 4.188) 0.0019

*Adjusted for multiple covariates (age, sex, race/ethnicity, BMI, education level, marital status, history of malignancy, diabetes mellitus, hypertension, smoking history, statins use, antihypertensive drugs, antidiabetic drugs, antiplatelet drugs, glucose, total cholesterol, diastolic blood pressure, systolic blood pressure, HbA1c (%), ALT; HDL-C; C reactive protein, blood urea nitrogen, and uric acid), excluding the stratification variable in each subgroup analysis.

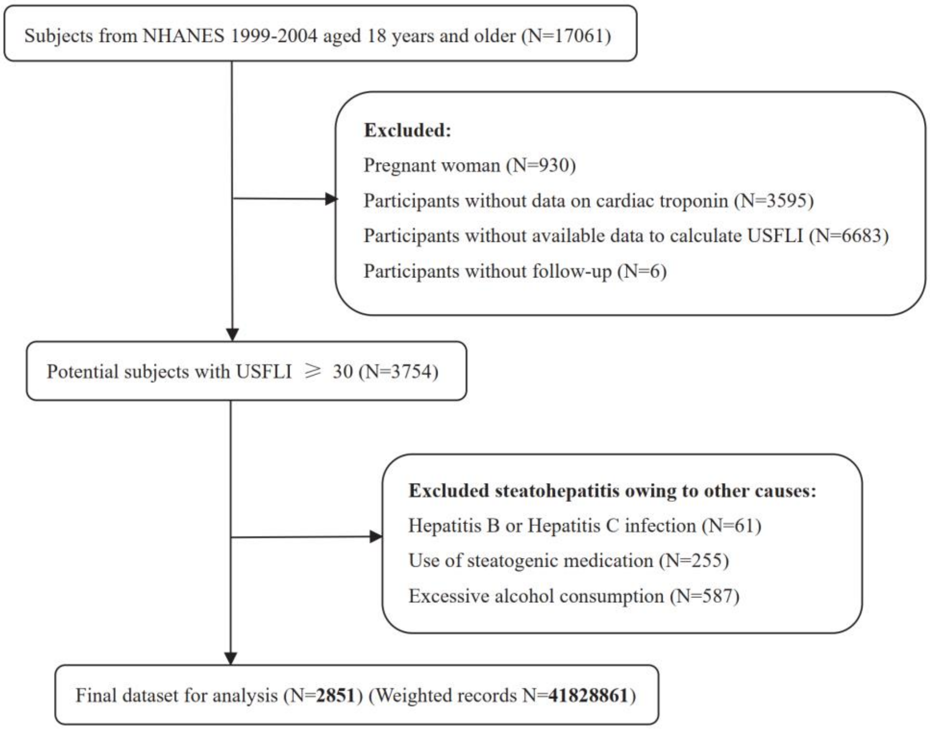

Supplementary Figure S1 Flowchart illustrating the selection process of the study participants

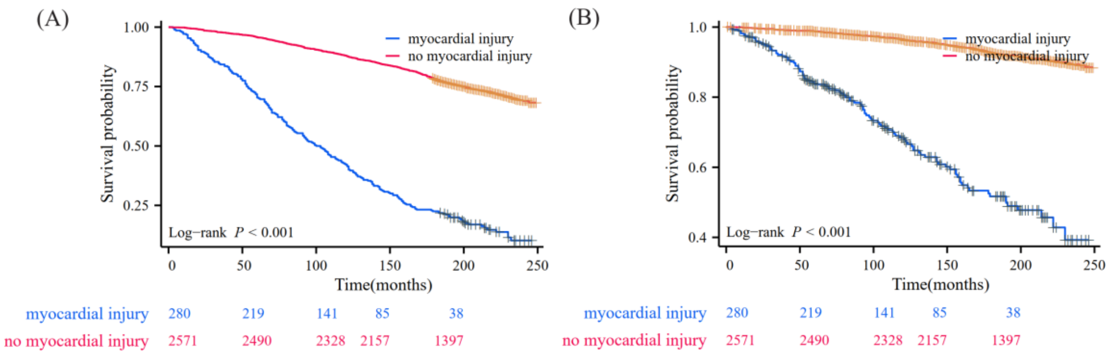

Supplementary Figure S2 Kaplan-Meier survival curve for patients with non-alcoholic fatty liver disease using hs-cTn (high-sensitivity cardiac troponin) T assay to define myocardial injury (Log-rank p < 0.001). (A) all-cause mortality; (B) cardiovascular disease mortality;

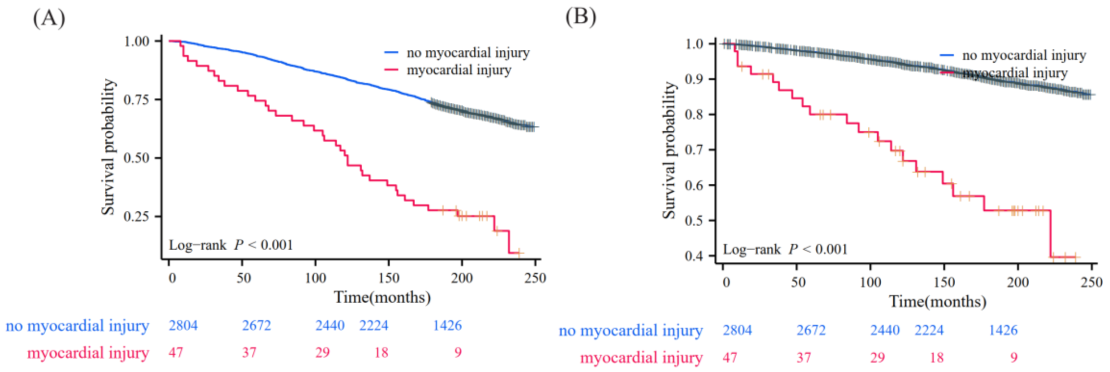

Supplementary Figure S3 Kaplan-Meier survival curve for patients with non-alcoholic fatty liver disease using Abbott hs-cTn (high-sensitivity cardiac troponin) T assay to define myocardial injury (Log-rank p <0.001). (A) all-cause mortality; (B) cardiovascular disease mortality;

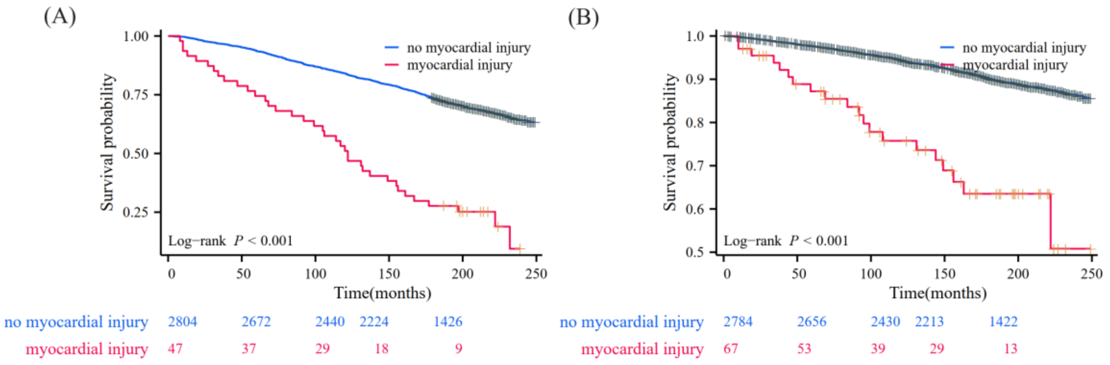

Supplementary Figure S4 Kaplan-Meier survival curve for patients with non-alcoholic fatty liver disease using Siemens hs-cTn (high-sensitivity cardiac troponin) T assay to define myocardial injury (Log-rank p <0.001). (A) all-cause mortality; (B) cardiovascular disease mortality;

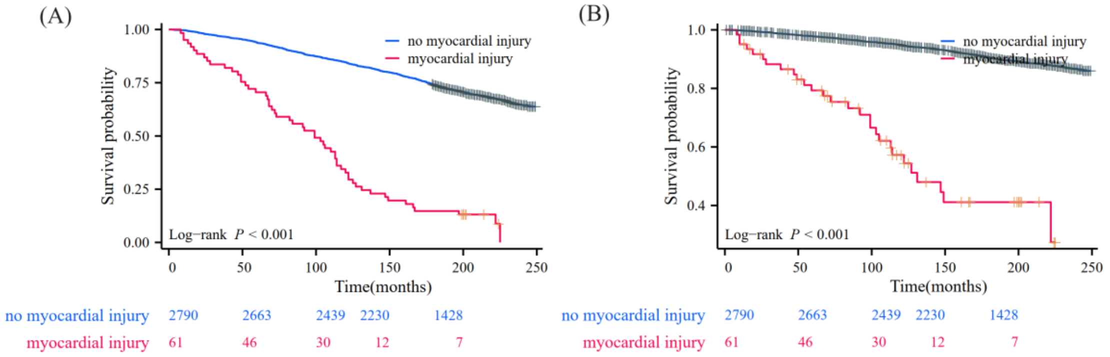

Supplementary Figure S5 Kaplan-Meier survival curve for patients with non-alcoholic fatty liver disease using Ortho hs-cTn (high-sensitivity cardiac troponin) T assay to define myocardial injury (Log-rank p <0.001). (A) all-cause mortality; (B) cardiovascular disease mortality;

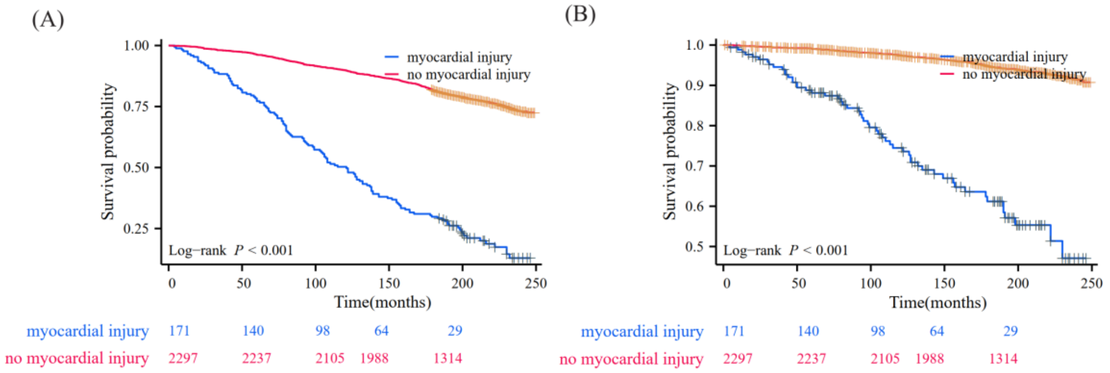

Supplementary Figure S6 Kaplan-Meier survival curve for patients with non-alcoholic fatty liver disease without known cardiovascular disease using hs-cTn (high-sensitivity cardiac troponin) T assay to define myocardial injury (Log-rank p <0.001). (A) all-cause mortality; (B) cardiovascular disease mortality;

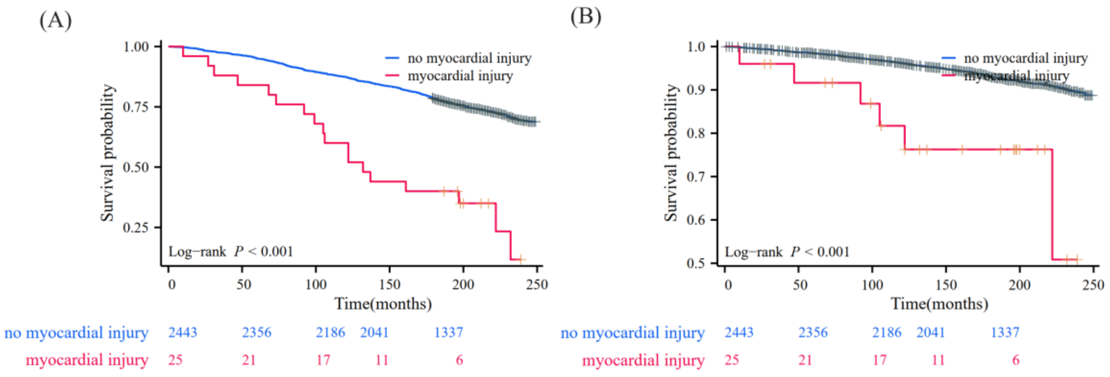

Supplementary Figure S7 Kaplan-Meier survival curve for patients with non-alcoholic fatty liver disease without known cardiovascular disease using hs-cTn (high-sensitivity cardiac troponin) I Abbott assay to define myocardial injury (Log-rank p <0.001). (A) all-cause mortality; (B) cardiovascular disease mortality;

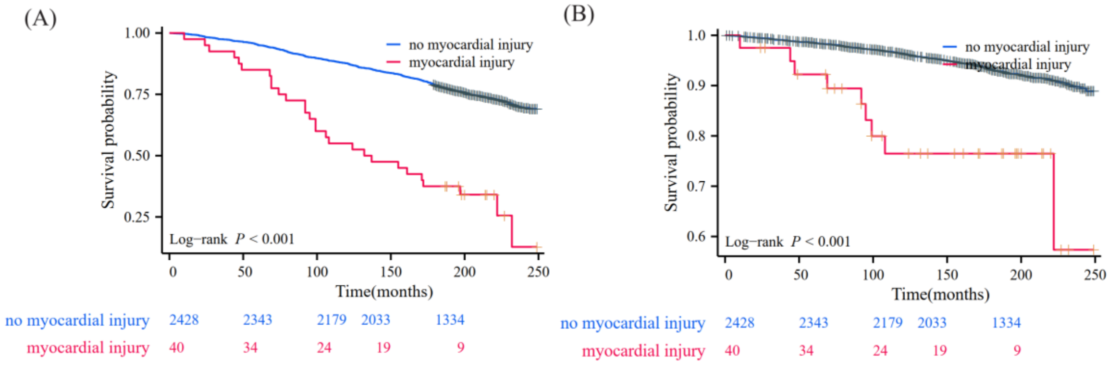

Supplementary Figure S8 Kaplan-Meier survival curve for patients with non-alcoholic fatty liver disease without known cardiovascular disease using hs-cTn (high-sensitivity cardiac troponin) I Siemens assay to define myocardial injury (Log-rank p <0.001). (A) all-cause mortality; (B) cardiovascular disease mortality;

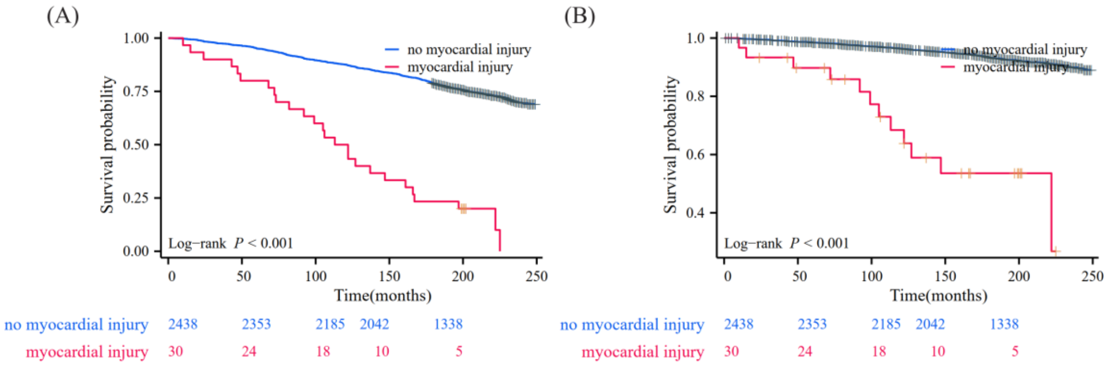

Supplementary Figure S9 Kaplan-Meier survival curve for patients with non-alcoholic fatty liver disease without known cardiovascular disease using hs-cTn (high-sensitivity cardiac troponin) I Ortho assay to define myocardial injury (Log-rank p <0.001). (A) all-cause mortality; (B) cardiovascular disease mortality;
